# Impact of renal replacement therapy strategy on beta-lactam plasma concentrations: the BETAKIKI study—an ancillary study of a randomized controlled trial

**DOI:** 10.1186/s13613-023-01105-0

**Published:** 2023-02-25

**Authors:** Damien Roux, Nicolas Benichou, David Hajage, Laurent Martin-Lefèvre, Nicolas de Prost, Nicolas Lerolle, Dimitri Titeca-Beauport, Eric Boulet, Julien Mayaux, Bruno Mégarbane, Khaoula Mahjoub, Dorothée Carpentier, Saad Nseir, Florence Tubach, Jean-Damien Ricard, Didier Dreyfuss, Stéphane Gaudry, for the AKIKI Study group

**Affiliations:** 1 DMU ESPRIT, Service de Médecine Intensive Réanimation, AP-HP, Université Paris Cité, Hôpital Louis Mourier, 92700 Colombes, France; 2 Université Paris Cité, INSERM, CNRS, Institut Necker Enfants Malades, 75015 Paris, France; 3 Sorbonne Université, INSERM Unit S_1155 CORAKID, 75010 Paris, France; 4 Département de Santé Publique, Centre de Pharmacoépidémiologie (Céphépi), Unité de Recherche Clinique PSL-CFX, Sorbonne Université, INSERM, Institut Pierre Louis d’Epidémiologie et de Santé Publique, AP-HP, Hôpital Pitié Salpêtrière, CIC-1901 Paris, France; 5 Réanimation Polyvalente, Centre Hospitalier Départemental - Site de La Roche-Sur-Yon, La Roche-sur-Yon, France; 6 Organ Donation Service, Centre Hospitalier Universitaire de Nantes, Nantes, France; 7 Réanimation Médicale, AP-HP, Hôpital Henri Mondor, Créteil, France; 8 Groupe de Recherche CARMAS, Université Paris-Est Créteil Val de Marne, 27010 Créteil, France; 9 Département de Réanimation Médicale et Médecine Hyperbare, CHU Angers, Université d’Angers, Angers, France; 10 BoRealStudy Group, Medical Intensive Care Unit and EA7517, Amiens University Hospital, 80054 Amiens, France; 11 Val d’Oise, Hôpital René Dubos, Pontoise, France; 12 Médecine Intensive Réanimation, AP-HP, Hôpital Pitié-Salpétrière, 75013 Paris, France; 13 Department of Medical and Toxicological Critical Care, Lariboisière Hospital, Université Paris Cité, Paris, France; 14 INSERM, UMRS-1144, Université Paris Cité, Paris, France; 15 Service de Réanimation, Hôpital Delafontaine, 93200 Saint-Denis, France; 16 Médecine Intensive Réanimation, Centre Hospitalier Universitaire Rouen, Rouen, France; 17 Centre Médecine Intensive-Réanimation, CHU de Lille and INSERM U1285, Université de Lille, CNRS, UMR 8576-UGSF, 59000 Lille, France; 18 Unité de Recherche Clinique, INSERM, UMR 1123, Paris, France; 19 Université Paris Cité, UMR1137 IAME, INSERM, 75018 Paris, France; 20 Médecine Intensive-Réanimation, AP-HP, Hôpital Avicenne, 93000 Bobigny, France

**Keywords:** Acute kidney injury, Beta-lactam, Concentration, Septic shock, Renal replacement therapy, Antibiotic, Intermittent hemodialysis, Hemofiltration

## Abstract

**Background:**

Sepsis prognosis correlates with antibiotic adequacy at the early phase. This adequacy is dependent on antibacterial spectrum, bacterial resistance profile and antibiotic dosage. Optimal efficacy of beta-lactams mandates concentrations above the minimal inhibitory concentration (MIC) of the targeted bacteria for the longest time possible over the day. Septic acute kidney injury (AKI) is the most common AKI syndrome in ICU and often mandates renal replacement therapy (RRT) initiation. Both severe AKI and RRT may increase outside target antibiotic concentrations and ultimately alter patient’s prognosis.

**Patients and methods:**

This is a secondary analysis of a randomized controlled trial that compared an early RRT initiation strategy with a delayed one in 620 critically ill patients undergoing severe AKI (defined by KDIGO 3). We compared beta-lactam trough concentrations between the two RRT initiation strategies. The primary outcome was the proportion of patients with sufficient trough plasma concentration of beta-lactams defined by trough concentration above 4 times the MIC. We hypothesized that early initiation of RRT could be associated with an insufficient antibiotic plasma trough concentration compared to patients allocated to the delayed strategy.

**Results:**

One hundred and twelve patients were included: 53 in the early group and 59 in the delayed group. Eighty-three patients (74%) had septic shock on inclusion. Trough beta-lactam plasma concentration was above 4 times the MIC breakpoint in 80.4% (*n* = 90) of patients of the whole population, without differences between the early and the delayed groups (79.2% vs. 81.4%, respectively, *p* = 0.78). On multivariate analysis, the presence of septic shock and a higher mean arterial pressure were significantly associated with a greater probability of adequate antibiotic trough concentration [OR 3.95 (1.14;13.64), *p* = 0.029 and OR 1.05 (1.01;1.10), *p* = 0.013, respectively). Evolution of procalcitonin level and catecholamine-free days as well as mortality did not differ whether beta-lactam trough concentration was above 4 times the MIC or not.

**Conclusions:**

In this secondary analysis of a randomized controlled trial, renal replacement therapy initiation strategy did not significantly influence plasma trough concentrations of beta-lactams in ICU patients with severe AKI. Presence of septic shock on inclusion was the main variable associated with a sufficient beta-lactam concentration.

*Trial registration*: The AKIKI trial was registered on ClinicalTrials.gov (Identifier: NCT01932190) before the inclusion of the first patient.

**Supplementary Information:**

The online version contains supplementary material available at 10.1186/s13613-023-01105-0.

## Background

Sepsis is a leading cause for Intensive Care Unit (ICU) admission. Empirical administration of an antibiotic of the beta-lactam class with broad antibacterial activity is recommended as initial treatment pending culture results [1]. Prognosis is indeed associated with antibiotic adequacy at the early phase [2, 3]. Reevaluation and possible de-escalation of treatment is recommended and usually done after the first 48 to 72 h. Adequacy of the antibiotic regimen is not only dependent on the antibacterial spectrum (or on the antibiotic resistance of the bacteria) of the antibiotic, but also on its dosage in order to attain sufficient tissue concentrations [1, 4]. Due to their wide spectrum, beta-lactams are part of empirical antibiotic therapy in ICU patients in most cases [1].

Beta-lactams are time-dependent antibiotics. Optimal efficacy mandates that their concentrations be above the minimal inhibitory concentration (MIC) of the targeted bacteria for the longest time possible over the day (optimally 24 h/day) in the infected organ. Delay in antibiotic adequacy (wrong molecule or insufficient dosage) results in increased short-term mortality [3, 5, 6]. Since it is not possible to evaluate actual tissue concentrations of beta-lactams in patients, it is admitted that a plasma trough concentration four times above MIC will assure sufficient beta-lactam concentration at the site of infection (except for neuro-meningeal infection which mandates higher doses) [7].

Prediction of antibiotic plasma concentration is challenging in critically ill patients as they often present with multi-organ failure including acute kidney injury (AKI) and/or altered hepatic function, and increased volume of antibiotic distribution [8]. Severe AKI frequently complicates sepsis and increases the challenge of the adaptation of antibiotic dosage. However, lowering beta-lactam dosage at an early phase because of fear of accumulation due to AKI may result in insufficient trough plasma concentration and exposes to the risk of treatment failure. In addition, renal replacement therapy (RRT) that is often required for severe AKI results in a clearance of the beta-lactam agent that is unpredictable. This may contribute to inadequate antibiotic concentration [9] and may affect survival.

The AKIKI study was a French multicenter randomized controlled study comparing two strategies for RRT initiation in patients with severe AKI (Stage 3 of KDIGO classification) and at least one other organ failure (invasive mechanical ventilation and/or catecholamine infusion) [10]. Patients were randomized to either receive immediate RRT or to a delayed strategy where RRT was initiated only when potentially severe complications developed (noticeable hyperkaliemia or metabolic acidosis or pulmonary edema) or when oliguria/anuria lasted for more than 72 h.

In this ancillary study of the AKIKI trial, we hypothesized that early initiation of RRT could result in an insufficient antibiotic plasma trough concentration compared to patients allocated to the delayed strategy group.

## Methods

The BETAKIKI study was a prospective observational study among patients included in the AKIKI trial. The AKIKI trial was an institutionally sponsored, prospective, multicenter, open-label randomized study conducted in 31 units in France between 2013 and 2016 [10]. All patients included in the AKIKI trial who received a beta-lactam for presumed or proved sepsis on inclusion were included in the BETAKIKI study in a subset of 10 participating centers. The AKIKI trial including the BETAKIKI ancillary study was approved by the ethics committee of the French intensive care society and the French legal authority (Comité de Protection des Personnes d’Ile de France VI). The investigators informed patients or their surrogates about the AKIKI trial and the BETAKIKI ancillary study both verbally and with a written document. In accordance with French law, written informed consent was not required, because the standard of care encompasses both study interventions. However, patients or their surrogates were informed that they could decline to participate at any time, and their decision was recorded in patient files.

### Selection–inclusion–randomization of subjects

Patients included in the AKIKI trial in the 10 participating centers were eligible to the BETAKIKI ancillary study if they were receiving a beta-lactam therapy at the time of randomization. Eligibility criteria for the AKIKI study were detailed in a previous publication and are displayed in the Additional file, [10]. The 1:1 randomization was computer-generated, stratified according to center with variable block sizes (CleanWeb, Telemedicine Technologies).

### Beta-lactam dosage

Choice and dosage of beta-lactam were left at physician’s discretion. Beta-lactam trough concentration was determined within 24 h after the randomization. In patients who did not receive immediate RRT (delayed strategy group in AKIKI), the serum level of antibiotic was determined on the morning following inclusion, just before a repeat injection of the beta-lactam. In case of continuous venous administration of the antibiotic, blood was sampled during regular biological sampling (morning time). Patients allocated to the early RRT initiation strategy underwent their first RRT session within a median of 2 [1–3] hours after randomization. They could receive continuous or intermittent RRT. In the former case, blood was drawn just before a repeat beta-lactam administration or during the regular blood sampling (morning time) in case of continuous antibiotic infusion. Patients receiving intermittent hemodialysis had the antibiotic level determination performed just before initiation of the second RRT session (about 24 h after randomization, see Additional file 1: Fig. S1). Plasma antibiotic level was again determined 24 h later based on the same procedures in all patients.

Beta-lactam level was measured routinely in the pharmacology laboratory at each participating centers.

### Assessment of beta-lactam efficacy

As previously proposed and because actual MIC of the targeted bacteria is unknown at the time of antibiotic initial administration, a trough plasma concentration was defined as sufficient if above four times the MIC breakpoint of susceptibility of the bacteria which was eventually isolated using the EUCAST recommendations (www.eucast.org, see Additional file 4: Table S1) [7]. If the serum level of antibiotic was not sufficient on at least one of the two determinations, it was considered inadequate. For *Pseudomonas aeruginosa*, the specific breakpoint of this bacterium was used (www.eucast.org).

We also considered procalcitonin serum level and number of catecholamine-free days as indirect clue to control of the infectious process. Procalcitonin serum level was determined at the time of randomization and on day 3 (24 h after the second beta-lactam level determination). The number of catecholamine-free days was assessed between randomization and day 28. Day 60 mortality was noted.

### Modification of the beta-lactam therapy during the study

If another beta-lactam was substituted for the initial one during the first 48 h, only that administered at the time of sampling was measured.

### Regimen of the beta-lactam therapy

We normalized the beta-lactam regimen by computing the ratio between the regimen received by the patient and the recommended one in stable patients (for instance cefotaxime 3 g per 24 h is the usual regimen in a patient without AKI. If the patient would receive 2 g/day, then this ratio would be 0.67). This allowed comparison of the two groups whatever the individual beta-lactam therapy.

### Outcomes

The primary outcome was the proportion of patients with sufficient trough plasma concentration of beta-lactams during the first 48 h post-randomization. The main objective was to compare this proportion between the two groups of patients: early RRT strategy group or delayed RRT strategy group. Patients in the latter group may have received RRT or not by protocol (i.e., they might have not reached the pre-specified criterion for RRT initiation).

We also compared patients with adequate beta-lactam concentrations and those without on the following criteria: (i) the factors associated with a sufficient beta-lactam concentration; (ii) the procalcitonin level evolution between randomization and day 3; (iii) the number of catecholamine-free days; (iv) day 60 mortality; and (v) the prescribed beta-lactam regimen by the physician.

### Statistics

Categorical variables were described as frequencies and percentages and were compared with the use of the Chi-square test or Fisher’s exact test, as appropriate. Quantitative variables were described as means and standard deviations or medians and interquartile ranges and were compared with the use of Student’s t-test or Wilcoxon test as appropriate.

Factors associated with sufficient beta-lactam concentration were determined using a univariate analysis followed by a multivariate analysis. Factors associated (*p* < 0.05) with adequate concentration in the univariate analysis were included in a multivariate logistic regression model. No further variable selection was performed. Because missing data accounted for less than 10% of patients for these candidate variables, analyses were performed on complete cases. Log linearity was graphically assessed for continuous variables using restricted cubic splines. Odds ratios (OR) and their 95% confidence interval (CI) were provided. Kaplan–Meier overall survival until Day 60 was estimated among alive patients after 48 h, and compared between adequate and inadequate groups using a logrank test. Significance was defined as p < 0.05. All statistical tests were two-sided. Statistical analyses were performed using R 3.5.1 (http://www.R-project.org).

## Results

Among the 234 patients included in the AKIKI trial in the 10 centers participating to the BETAKI study, 112 were included in this study: 53 in the early strategy group and 59 in the delayed strategy group (Additional file 2: Figure S2). Table 1 describes patient characteristics at baseline. Eighty-three patients (74%) had septic shock on inclusion.

**Table 1:** Baseline characteristics of patients

	Early strategy (*N* = 53)	Delayed strategy (*N* = 59)
Sex–*N*, % male	41 (77.4)	35 (59.3)
Age–year	67 [55–73]	66 [58–76]
Underlying disease, *N* (%)		
- Chronic kidney disease^a^	3 (5.7)	4 (6.8)
- Chronic arterial hypertension	29 (54.7)	29 (49.2)
- Congestive heart failure	6 (11.3)	4 (6.8)
- Ischemic heart disease	6 (11.3)	5 (8.5)
- Chronic liver disease	1 (1.9)	1 (1.7)
- Diabetes mellitus	15 (28)	10 (17)
- Malignant disease	10 (18.9)	14 (23.7)
- Neutropenia	1 (1.9)	3 (5.1)
SAPS III^b^ at enrollment	61 [55–73]	67 [58–74]
SOFA^c^ score at enrollment	11 [9–13]	10 [8–13]
Physiological support, *N* (%)		
- Invasive mechanical ventilation	48 (90.6)	52 (88.1)
- Vasopressors	44 (83.0)	53 (89.8)
Sepsis status, *N* (%)		
- Septic shock	38 (71.7)	45 (76.3)
- Severe sepsis	2 (3.8)	5 (8.5)
- Sepsis	2 (3.8)	4 (6.8)
- No sepsis	11 (20.8)	5 (8.5)
Serum creatinine, µM	192 ± 137	186 ± 120
Serum urea, mM	12.9 ± 9.1	14.4 ± 9.8
Patients with oliguria or anuria, *N* (%)	31 (59.6)	25 (43.9)

^a^Chronic kidney disease: eGFR ≤ 60 ml/min

^b^The Simplified Acute Physiology Score (SAPS) III ranges from 0 to 146, with higher scores indicating more severe dis‐ ease and a higher risk of death

^c^The Sepsis‐related Organ Failure Assessment (SOFA) score ranges from 0 to 24, with higher scores indicating more severe organ failure

All patients in the early strategy group received RRT, as expected. In the delayed strategy group, 41 (69%) patients did not receive RRT at the time of sampling. Five were receiving RRT at the time of first sampling and 13 at the time of second sampling. Cefotaxime (33%), ceftriaxone (14%) and piperacillin–tazobactam (21%) were the most frequently used beta-lactam agents (Table 2). The primary site of infection was mainly bloodstream infection (26%), pneumonia (21%) and intra-abdominal sepsis (17%) (Table 2).

**Table 2:** Type of beta-lactam therapy, site of infection and microorganisms

	Total (*N* = 112)
Type of Beta-lactam^a^	
• Amoxicillin	11
• Amoxicillin + clavulanate	7
• Cloxacillin	6
• Piperacillin	4
• Piperacillin + tazobactam	23
• Cefotaxime	37
• Ceftriaxone	16
• Ceftazidime	3
• Imipenem	10
Site of infection	
• Bloodstream infection	29
• Pneumonia	23
• Abdominal infection	19
• Urinary tract infection	11
• Meningitis	4
• Skin and soft tissue infection	8
• Other	5
• None	13
Identified microorganisms^b^	
Gram positive	
• *Streptococcus pneumoniae*	6
• Other* Streptococcus*	7
• *Staphylococcus aureus*	12
• Coagulase negative* Staphylococcus*	4
• *Enterococcus* sp.	6
Gram negative	
• *Haemophilus* sp.	3
• *Escherichia coli*	27
• *Klebsiella pneumoniae*	14
• Other *Enterobacteriaceae*	16
• *Pseudomonas aeruginosa*	8
• *Legionella pneumophila*	3
• Other	6

^a^Some patients received 2 beta-lactam agents

^b^Some patients had up to 4 identified pathogens

### Primary outcome

**Fig. 1:**
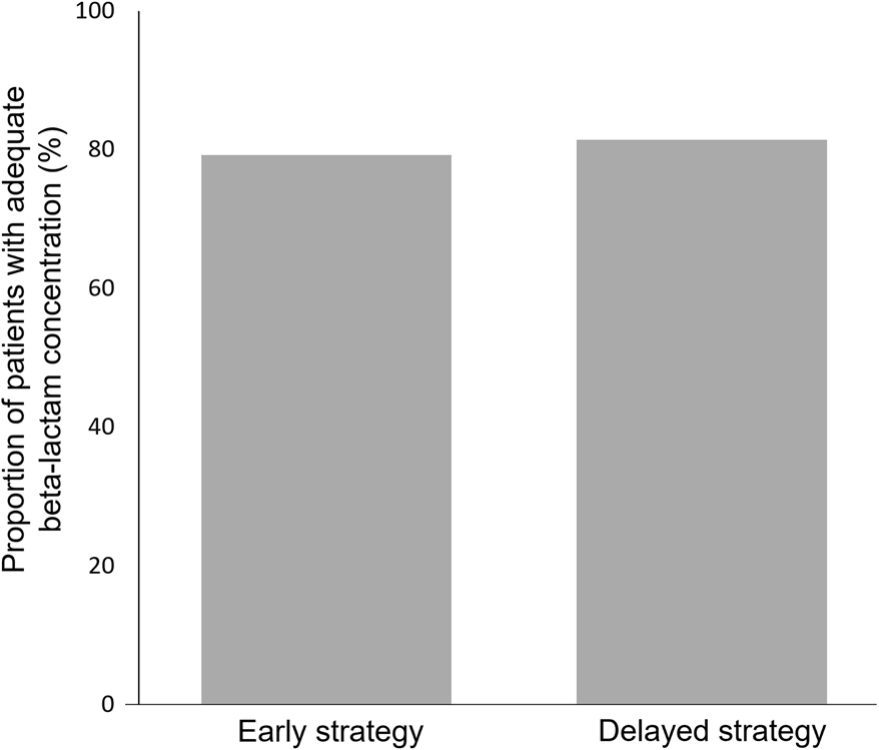
Proportion of patients with adequate trough beta-lactam plasma concentration in early and delayed strategy group

Trough beta-lactam plasma concentration was above 4 times the MIC breakpoint in 80.4% (*n* = 90) of patients of the whole population. The two groups did not differ with respect to the adequacy of plasma concentration. Indeed, trough beta-lactam plasma concentration was above 4 times the MIC breakpoint in 79.2% (*n* = 42) in the early strategy group and 81.4% (*n* = 48) in the delayed strategy group (*p* = 0.78, Fig. 1).

### Secondary outcomes

The following analyses were performed between patients with adequate beta-lactam concentrations and those without.

- *Factors associated with adequate beta-lactam trough concentration*
  On univariate analysis, presence of a septic shock, higher mean arterial pressure, higher creatinine plasma level, lower bicarbonate plasma level and recent administration of an aminoglycoside were significantly associated with adequate beta-lactam concentrations (Table 3). On multivariate analysis, the presence of septic shock and a higher mean arterial pressure were significantly associated with a greater probability of adequate antibiotic trough concentration [OR 3.95 (1.14;13.64), *p* = 0.029 and OR 1.05 (1.01;1.10), *p* = 0.013, respectively, Table 3].
- *Procalcitonin serum level*
  At inclusion, procalcitonin serum levels were significantly higher in patients with adequate beta-lactam concentrations as compared to others. Procalcitonin serum levels decreased in both groups and its evolution between the time of inclusion and day 3 did not significantly differ whether beta-lactam trough concentration was above 4 times the MIC or not (Additional file 4: Table S1).
- *Catecholamine-free days*
  The number of catecholamine-free days did not significantly differ between patients with sufficient beta-lactam trough concentration as compared to patients with inadequate dosage [21 (10–26) vs. 25 (9–26), respectively, *p* = 0.47].
- *Mortality*
  Day-60 mortality did not differ whether beta-lactam trough concentration was adequate or not (*p* = 0.81, Additional file 3: Figure S3).
- *Beta-lactam prescription and beta-lactam trough concentrations*
  Using the normalized variable representing the beta-lactam regimen, we observed that patients with adequate trough concentrations received higher dose of beta-lactam as compared to patients with lower trough concentrations (normalized regimen per 24 h between inclusion and first dosage were, respectively, 1.23 (± 0.61) and 0.75 (± 0.31), *p* = 0.03). Adequate plasma concentrations of antibiotics were observed when drug regimen was above the regular regimen recommended in stable patients. (Additional file 4: Table S2).

**Table 3:** Factors associated with adequate beta-lactam concentration (> 4 times the MIC threshold)

			Univariate analysis	Multivariate analysis	
	Inadequate (*N* = 22)	Adequate (*N* = 90)	*p* value	OR [CI 95%]	*p* value
Sex (female), *N* (%)	7 (31.8)	29 (32.2)	0.97		
Age (year), mean (SD)	67.0 (10.0)	64.3 (13.7)	0.57		
Weight (kg), mean (SD)	84.5 (12.8)	86.1 (20.9)	0.67		
Delay between ICU admission and randomization, median [IQ]	1.5 [1–4.75]	1 [1, 2]			
Chronic kidney disease, N (%)	0 (0)	7 (7.8)	0.34		
Delayed strategy group (AKIKI randomization), *N* (%)	11 (50)	48 (53.3%)	0.78	1.18 [0.40–3.45]	0.767
SAPS III, mean (SD)	70.0 (8.4)^a^	70.7 (14.4)^b^	0.97		
Administration of aminoglycosides	4/14 (28.6%)	33/56 (58.9%)	0.04	2.24 [0.58–8.72]	0.23
Administration of vancomycin	1/14 (7.1%)	11/56 (19.6%)	0.43		
Septic shock, *N* (%)	10 (45.5)	73 (81.1)	0.0006	**3.95 [1.14–13.64]**	**0.029**
Mean arterial pressure (mmHg), mean (SD)	70 (7)	77 (16)	0.0181	**1.05 [1.01–1.10]**	**0.013**
Cardiac rate (beats per minute), mean (SD)	99 (24)	111 (28)	0.053		
Serum creatinine (µM), mean (SD)	231.3 (100.8)	289.6 (112.5)	0.024	1.01 [1.00–1.01]	0.062
Serum urea (mM), mean (SD)	17.6 (8.2)	18.5 (8.0)	0.82		
Serum bicarbonate (mM), mean (SD)	21.3 (5.6)	19.0 (4.4)^a^	0.045	0.97 [0.86–1.09]	0.585
Arterial blood lactate (mM), mean (SD)	2.5 (1.8)^c^	2.7 (2.4)^d^	0.98		

Bold values specifies the statistically significant variables in the multivariate analysis

^a^1 missing data

^b^6 missing data

^c^3 missing data

^d^8 missing data

## Discussion

This prospective observational ancillary study of the randomized controlled trial AKIKI aimed at evaluating beta-lactam plasma trough concentrations in critically ill patients with severe AKI according to the strategy of RRT initiation. We did not observe significant difference in beta-lactam plasma trough concentrations in the 2 days following randomization according to RRT initiation strategy.

Antibiotic dosing in critically ill patients with AKI may be a hardship. Indeed, toxic levels may be attained in patients who do not receive RRT, whereas RRT may lead to suboptimal levels. Our study shows that provided adequate rules for dosing are applied, the levels are acceptable in both cases in the majority of patients. The AKIKI trial compared two RRT initiation strategies in patients with severe (KDIGO3 stage) AKI: an early one where RRT was immediately started and a delayed one where RRT was postponed until some criteria (duration of anuria, a certain level of uremia among others, metabolic complication) were reached [10]. With the delayed strategy, 49% did not receive RRT by the end of the AKIKI study. Those who received it had it started 57 h after randomization. In the present BETAKIKI study, all patients allocated to the early RRT strategy were receiving this treatment at the time of antibiotic administration. In the delayed strategy group 13 of the 59 patients received RRT in the 48 h following inclusion. Then, the vast majority of patients allocated to the delayed strategy were not receiving RRT at the time of this study.

Because clinical evolution and infection control in such patients are dependent of multiple parameters among which adequacy of antibiotic treatment, our study targeted plasma antibiotic concentration instead of clinical or microbiological parameters. We observed that more than 80% of patients had a beta-lactam trough concentration above 4 times the MIC breakpoint for the selected beta-lactam, irrespective of the RRT initiation strategy. In other words, the timing of RRT had no influence on the adequacy of beta-lactam trough concentrations. Since the BETAKIKI study was purely observational, this suggests that physicians correctly adapted beta-lactam regimen in order to avoid the risk of insufficient dosage, especially in patients with confirmed septic shock.

These findings probably highlight the progress achieved regarding antibiotic dosing for critically ill patients during the last years. Indeed, multiple reports from the late 2000’s showed frequent (more than 50%) insufficient beta-lactam trough concentrations in the ICU, especially in patients receiving CRRT [3, 11, 12].

Identification of factors associated with insufficient concentrations such as higher weight or total renal clearance [13], including residual renal function or CRRT intensity [14, 15], allows for more individualized antibiotic dosing [16]. To our knowledge, our study is the first to find an association between septic shock (identified as such by the treating physician) and sufficient beta-lactam concentration (odds ratio around 4). Although physiological parameters could have influenced this association, we hypothesize that it is mostly the consequence of higher regimen chosen by physicians in this situation, since we found a significant correlation between regimen and concentrations, and regimen used for patients with septic shock at admission seemed higher (albeit not reaching statistical significance).

This personalization of treatment has been recently observed in the international SMARRT trial, which prospectively studied 508 trough antibiotic concentrations in 381 critically ill patients with AKI requiring RRT [15]. One of the most striking findings from this study were the wide variability in dosing regimen used by physician (for instance variation of daily dose up to eightfold for meropenem), and in antibiotic concentrations. Interestingly, sufficient concentrations were not reached for at least 25% of patients, which is close to our results, in a similar population. This variability strengthens the need for easily available therapeutic drug monitoring [17], and the development of dosing softwares as promising areas to improve clinical prognosis of patients [18], which relies on adequate antibiotic concentrations [3].

There is no obvious explanation for the higher mean arterial pressure statistically associated with adequate antibiotic dosage. The absence of difference in procalcitonin serum level change over time and in catecholamine-free days whether the serum beta-lactam concentration was adequate or not probably reflected the numerous confounding factors present in critically ill patients. In addition, the use of four times the MIC breakpoint of EUCAST was a very conservative approach for defining a sufficient plasma trough concentration. Most susceptible bacteria had a much lower MIC for the beta-lactam than the MIC breakpoint. In other words, we elected to use a worst-case scenario which probably led to an overestimation of the number of instances were beta-lactam plasma concentration was deemed insufficient.

Our study has limitations. First, we determined serum beta-lactam concentration only twice 24 h apart at the early phase of the critical illness. This was because we chose a simple protocol in order to recruit a maximum number of patients. Noteworthy is the fact that the prognosis of septic shock is dependent on the precocity and adequacy of antibiotic administration [2, 5, 6]. Therefore, the first doses and their resulting serum concentrations are probably determinant in this context. Second, the serum beta-lactam concentration was determined in different laboratories, depending on the ICU location. This was part of routine care for these severe patients, and it represented real-life. Third, because we used the MIC EUCAST breakpoints instead of the actual MIC of each identified bacterium (determination of MIC was not routinely done for susceptible bacteria), we may have misclassified some patients as inadequate dosage, resulting in a worst-case scenario. The inverse (underestimating this number) would be much more problematic. Finally, RRT modalities and doses, beta-lactam mode of administration (continuous, prolonged or intermittent perfusion) or beta-lactam concentrations following the first 48 h were not evaluated.

In contrast, the main strength of our study is to demonstrate that RRT initiation strategy did not affect the potential efficacy of the antibiotic regimen in a real-life setting. This is a reassuring finding. Another strength of the study is that we used a clearly defined protocol for beta-lactam dosage according to RRT modality.

This study shows that provided clinicians apply theoretical rules for determination of antibiotic regimen, serum initial levels are adequate in infected patients with septic shock for the majority and with severe AKI irrespective of the RRT initiation strategy.

## Conclusions

Renal replacement therapy initiation strategy did not significantly influence plasma trough concentrations of beta-lactams in ICU patients with severe AKI. Presence of septic shock on inclusion was the main variable associated with a sufficient beta-lactam concentration.

## Supplementary Information

**Additional file 1: Figure S1.** Schematic presentation for timing of beta-lactam and procalcitonin dosages depending on strategy groups and methods of renal replacement therapy. Panel A - early strategy group with intermittent hemodialysis; panel B - early strategy group with continuous veno-venous hemofiltration or hemodiafiltration; panel C - delayed strategy group. If a patient in the delayed strategy group received a RRT session, the sampling followed panel A or B depending on the method used. PCT procalcitonin dosage; IHD intermittent hemodialysis; ATB antibiotic; RRT renal replacement therapy.

**Additional file 2: Figure S2.** Flow chart

**Additional file 3: Figure S3.** Kaplan–Meier overall survival until Day 60 of patients with and without adequate dosage of beta-lactams. Kaplan–Meier overall survival until Day 60 was estimated among patients alive after 48 hours and compared between adequate and inadequate group using a logrank test.

**Additional file 4: Table S1.** Procalcitonin serum levels and its evolution with or without adequate beta-lactam concentrations. **Table S2.** Beta lactam regimen and concentrations.

## Data Availability

The datasets used and/or analyzed during the current study are available from the corresponding author on reasonable request.

## Abbreviations

AKI: Acute kidney injury
CI: Confidence interval
CRRT: Continuous renal replacement therapy
EUCAST: European Committee on Antimicrobial Susceptibility Testing
ICU: Intensive care unit
KDIGO: Kidney Disease: Improving Global Outcomes
MCI: Minimal inhibitory concentration
OR: Odds ratio
RRT: Renal replacement therapy
