# Supplementary figures and images for: Impact of renal replacement therapy strategy on beta-lactam plasma concentrations: the BETAKIKI study—an ancillary study of a randomized controlled trial
Source: Ann Intensive Care. 2023 Feb 25;13:11. doi: 10.1186/s13613-023-01105-0 (PMC9968363; doi:10.1186/s13613-023-01105-0)

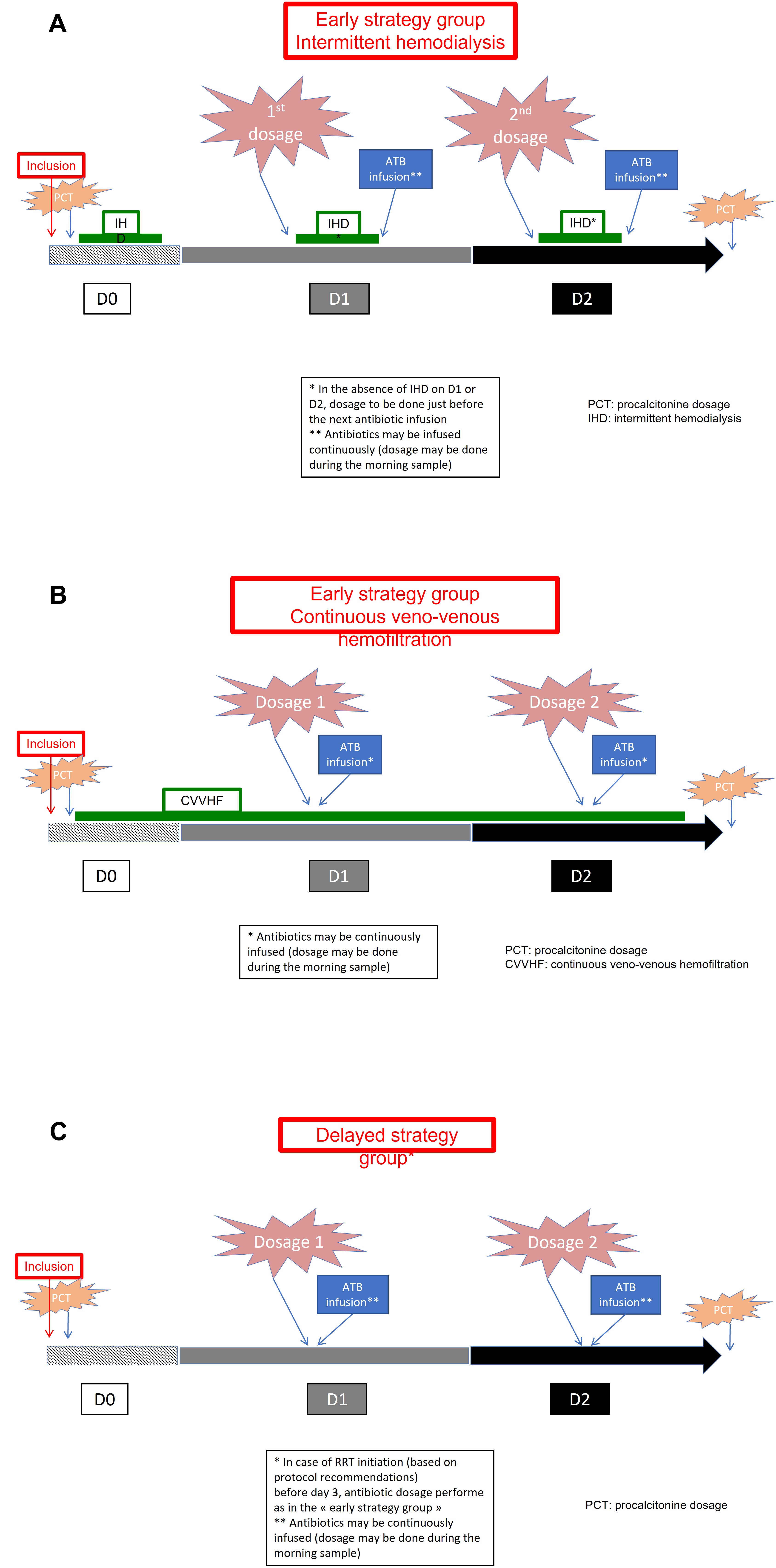

Supplement: Supplementary file 1 — Additional file 1: Figure S1. Schematic presentation for timing of beta-lactam and procalcitonin dosages depending on strategy groups and methods of renal replacement therapy. Panel A - early strategy group with intermittent hemodialysis; panel B - early strategy group with continuous veno-venous hemofiltration or hemodiafiltration; panel C - delayed strategy group. If a patient in the delayed strategy group received a RRT session, the sampling followed panel A or B depending on the method used. PCT procalcitonin dosage; IHD intermittent hemodialysis; ATB antibiotic; RRT renal replacement therapy. [file 13613_2023_1105_MOESM1_ESM.jpg]

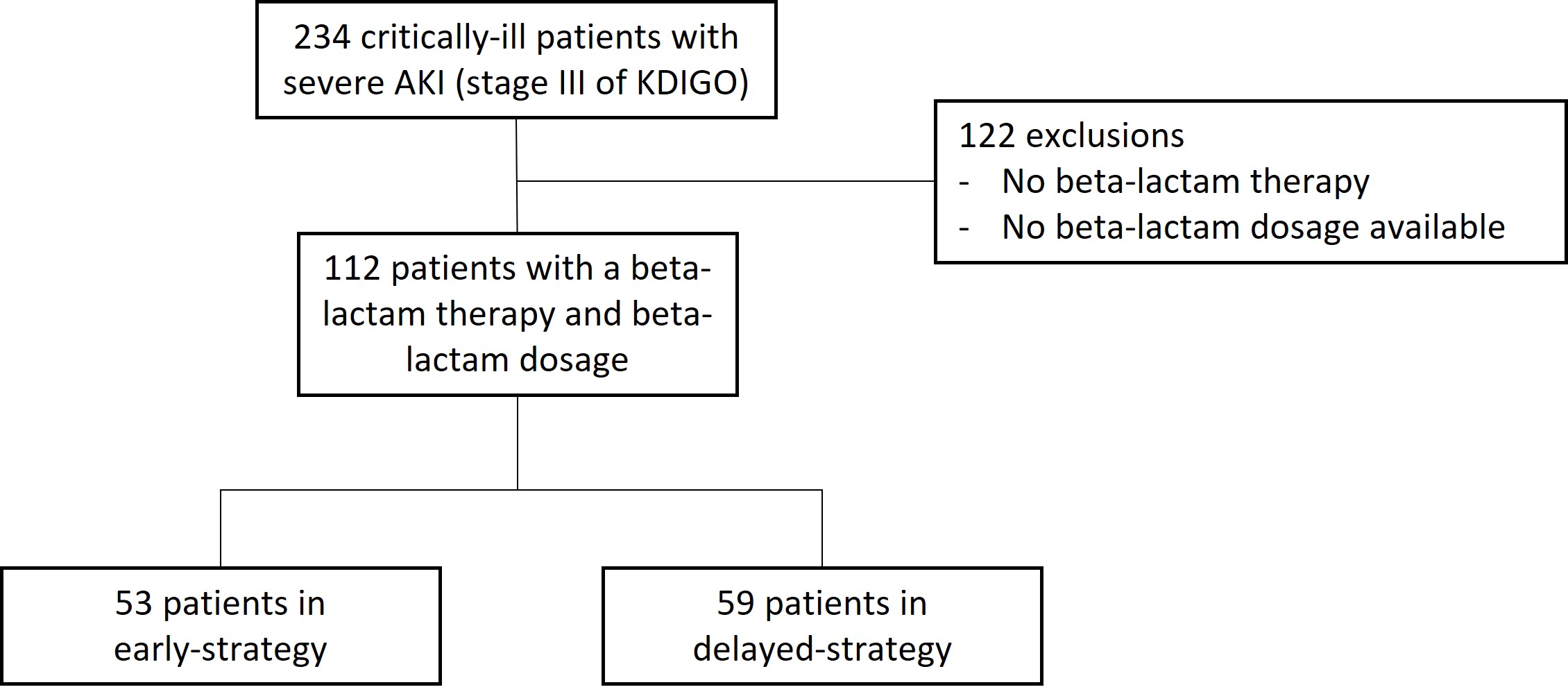

Supplement: Supplementary file 2 — Additional file 2: Figure S2. Flow chart [file 13613_2023_1105_MOESM2_ESM.jpg]

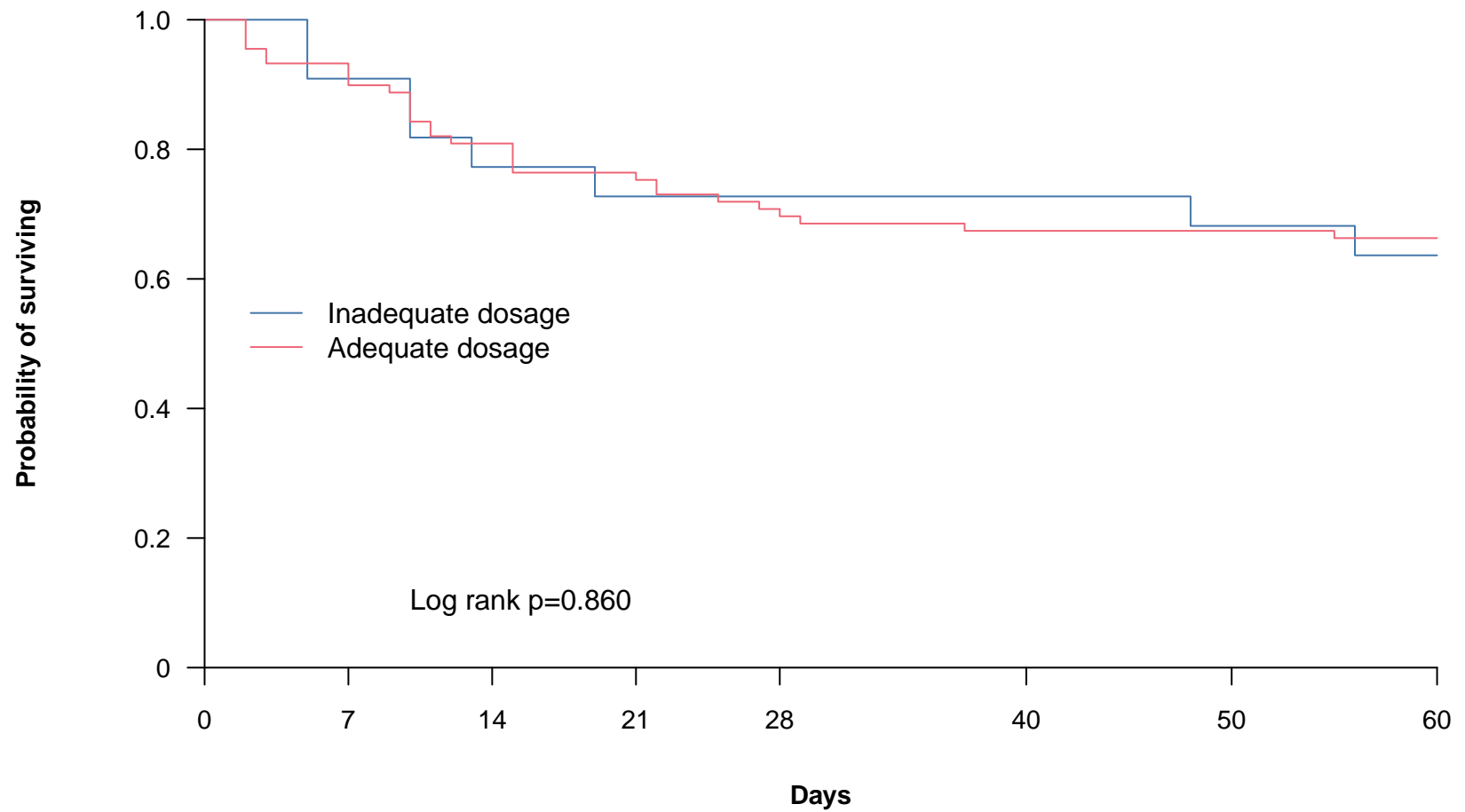

Number at risk (number censored)

|   |        |        |        |        |        |        |        |         |
|---|--------|--------|--------|--------|--------|--------|--------|---------|
| — | 22 (0) | 20 (0) | 17 (0) | 16 (0) | 16 (0) | 16 (0) | 15 (0) | 14 (14) |
| — | 89 (0) | 83 (0) | 72 (0) | 68 (0) | 63 (0) | 60 (0) | 60 (0) | 59 (59) |

Supplement: Supplementary file 3 — Additional file 3: Figure S3. Kaplan–Meier overall survival until Day 60 of patients with and without adequate dosage of beta-lactams. Kaplan–Meier overall survival until Day 60 was estimated among patients alive after 48 hours and compared between adequate and inadequate group using a logrank test. [file 13613_2023_1105_MOESM3_ESM.pdf]
