# Supplementary material for: Impact of renal replacement therapy strategy on beta-lactam plasma concentrations: the BETAKIKI study—an ancillary study of a randomized controlled trial
Source: Ann Intensive Care. 2023 Feb 25;13:11. doi: 10.1186/s13613-023-01105-0 (PMC9968363; doi:10.1186/s13613-023-01105-0)
Supplement: Supplementary file 4 — Additional file 4: Table S1. Procalcitonin serum levels and its evolution with or without adequate beta-lactam concentrations. Table S2. Beta lactam regimen and concentrations. [file 13613_2023_1105_MOESM4_ESM.docx]

**Table S1. Procalcitonin serum levels and its evolution with or without adequate beta-lactam concentrations**

|  | Inadequate trough concentration  (N=17) | Adequate (> 4 times the MIC threshold) trough concentration  (N=46) | p value |
| --- | --- | --- | --- |
| Procalcitonin at inclusion (ng/mL) | 35.8 [4.9;54.1] | 59.0 [16.7-142.7] | 0.01 |
| Procalcitonin at day 3 (ng/mL) | 8.9 [0.7;16.8] | 14.7 [2.6-40.9] | 0.08 |
| Procalcitonin variation (%) | -70.8 [-82.2;-50.0] | -67.5 [-75.5;-50.1] | 0.70 |

**Table S2. Beta lactam regimen and concentrations**

|  | Inadequate trough concentration at day 1 | Adequate (> 4 times the MIC threshold) trough concentration at day 1 | p value |
| --- | --- | --- | --- |
| Normalized beta lactam regimen per 24h, before inclusion ^1^  Mean (SD) | 0.97 (0.32)  (n = 9) | 1.31 (0.68)  (n = 47) | 0.21 |
| Normalized beta lactam regimen per 24h, between inclusion and first dosage ^1^  Mean (SD) | 0.75 (0.31)  (n = 8) | 1.23 (0.61)  (n = 45) | 0.03 |

^1^  Normalized beta-lactam regimen : numeric variable representing the ratio between the individual regimen received by the patients and the recommended regimen in stable patients, i.e. : Amoxicilline: 3g per 24h, Cefotaxime: 3g per 24h, Ceftriaxone: 1g/24h, Piperacilline (+/- Tazobactam): 16g per 24h if aiming at covering *Pseudomonas Aeruginosa*, 12g per 24h if not, Ceftazidime: 4g per 24h if aiming at covering *Pseudomonas Aeruginosa*, 3g per 24h if not, Imipenem and Meropenem: 3g per 24h, Cloxacilline: 8g per 24h
